# A Reflection on Controversial Literature on Screen Time and Educational Apps Use in 0–5 Years Old Children

**DOI:** 10.3390/ijerph17134641

**Published:** 2020-06-28

**Authors:** Luca Cerniglia, Silvia Cimino

**Affiliations:** 1 Faculty of Psychology, International Telematic University Uninettuno, Corso Vittorio Emanuele II, 39, 00186 Rome, Italy; 2 Dynamic and Clinical Department, Sapienza University of Rome, 00186 Rome, Italy; silvia.cimino@uniroma1.it

**Keywords:** screen time, educational apps, 0–5 children

## Abstract

Over the last five years, there has been a significant increase in screen time and apps usage by children under five years old. The considerable growth in usage by very young children has not corresponded to conclusive and consistent research investigating its possible benefits and risks. This article proposes a brief overview of recent results in this field, specifically focusing on the use of educational apps and their positive, null, and/or negative outcomes on young children’s cognitive, emotional, and behavioral functioning. The aim of the present article is to stimulate the development and advancement of evidence-based guidelines that caregivers and educators could adopt to regulate very young children’s engagement with digital technologies.

## 1. Introduction

A growing literature is focusing on the possible positive and negative outcomes associated with the use of technological devices and apps (played on smartphones and Ipads) in children in their first five years of life [1]. Apparently, two conflicting branches of literature are accumulating results in this field: (a) studies investigating whether the use of technology has an impact on learning and cognitive tasks (literacy, mathematics, science, etc.) [2]; and (b) studies concentrating on the possible effects on the emotional and behavioral functioning of children using touch screen devices (especially in the case of their excessive use) [3]. Interestingly, the first branch of literature seems to have gathered overall positive results of screen devices on children’s cognitive performances, while the second branch appears to have gathered evidence that screen time (especially below the age of 5 years) is problematic and should be reduced, if not avoided completely. These inconsistent results put caregivers and educators in doubt whether to propose the use of these technologies to young children, given that they seem to carry both positive outcomes and negative burdens.

On one hand, digital technology for young children is powerfully promoted (particularly by the industry) based on promising results of studies that showed enhanced learning capacities, inclusion, augmented engagement in STEM (i.e., science, technology, engineering, and mathematics), and higher productivity and competence in social interaction, thanks to an active use of smart devices, in collaboration with caregivers and educators. For example, e-books seem to be useful in stimulating vocabulary development and reading comprehension and are suggested to be more engaging for young children thanks to “digital scaffolds” (e.g., synchronous text highlighting, sound effects, animations) [4].

On the other hand, public health agencies support a minimal use of digital technology by children younger than five years, due to concerns about its negative effects on physical, cognitive, and emotional well-being, eventually leading to a general impairment in youths’ development [5]. Even research investigating correlations between screen time and emotional-behavioral functioning of older children and adolescents has been inconsistent; some studies found significant associations between screen time and low well-being [6,7], whereas other findings showed null effects of screen time or even positive outcomes [8,9,10].

With a balanced position, the American Academy of Pediatrics suggested that video chat and quality content is permissible for infants (as young as 0–5 years old) in the presence of a caregiver. However, in the case of children under five years of age, research has showed that the use of digital devices by young children is often passive and is not scaffolded by adults [11]. 

### 1.1. Physical, Cognitive, Emotional, and Social Concerns

Some research has shown that poor postures and limited and repetitive movements during the use of smart devices can possibly lead to sedentary routines influencing energy expenditure, eventually causing overweight and obesity [9]. As for the cognitive aspects, some scholars have warned about shortened attention spans, fewer occasions for verbal interactions, impaired problem-solving, and reduced creativity [3], whereas emotional concerns include addiction, anxiety and depression symptoms, and access to inappropriate content when unsupervised by an adult [12]. In the social realm, concerns comprise isolation, cyber-bullying, and predatory behaviors (that are more probable in younger children who are not capable of identifying potentially dangerous contents, although instructed by parents or caregivers [13]. Interestingly, it has been posited that parental engagement in children digital activities could limit unwanted exposure of children to inappropriate contents. However, it has been shown that children can nevertheless be confronted with inapt content, due to the easy-to-use graphic interfaces allowing them to activate other videos from the suggested playlist that is shown beside the original content set up by the adult [14]. Policy resources orientating automatic systems of protection have in fact been established having in mind older children (from 8 to 18 years), so little thought has been given to the security of young children using digital devices.

### 1.2. Educational Apps Are Fine, or Are They?

Apps could represent a meaningful chance for out-of-school, informal learning, if designed appropriately [15]. Some authors have posited that an “educational” app should promote a high level of interactivity (promoting an active role of the child in its use), increase the child’s familiarity with technology, be targeted to the child’s developmental phase, provide knowledge of results comprehensible for the child, and promote participation and collaboration among peers and with caregivers and educators [16]. If apps are designed with these characteristics, research has indicated that tablet use by preschoolers could promote drawing skills [17,18] and creative thinking [19], film-making, music creation, and photography [20,21]. Such well-constructed apps can increase young children’s school readiness, or executive function competence, and could have long-term effects [22]. However, the vast majority of these apps have no evidence for the claim of being effectively educational; in fact, it has been posited that they can make it more difficult for children to interact in face-to-face exchanges with adults and peers because smart devices tend to be used individually, and to eventually lead to an impairment in other important capacities, such as empathy and self-regulation [23]. Therefore, the guidelines of the American Academy of Pediatrics (that urges parents and caregivers not to expose young children to screen time or at least to propose to their offspring only high-quality educational apps [24] are almost impossible to follow for parents and educators because the vast majority of the apps targeted at young children were not created to be educational.

### 1.3. Is It Excessive Use the Only Problem?

The problem also seems to not just be connected to the passive or excessive use of these technologies; accumulating literature is demonstrating that the use in itself can be problematic (if not even dangerous) in very young children [25]. According to this position, screen time can have an effect on brain functioning causing a constellation of symptoms related to mood, anxiety, cognition, and behavior, which considered together form an emotional and behavioral dysregulation syndrome, especially when children are exposed to engaging apps (supposedly more adapted to their age because they are more stimulating and cognitively interesting). Due to over-stimulation, interactive screen-time puts the nervous system into fight-or-flight mode, causing dysregulation, disorganization, and distress [26]. It is very important to note that these negative outcomes in children can be immediately visible, for example, observing a child after he/she used very activating video games, or can take place slowly and progressively after years of recurring screen use [27]. 

## 2. Conclusions

It could be argued that today’s apps are part of the “first wave” of the digital revolution (e.g., apps reproducing in e-format-consolidated non-digital games and puzzles without explicitly considering young children’s learning processes and the unique affordances of digital media to support their learning). At any rate, most of the apps that are today used as “educational” do not have these requisites, although some effective proposals have been recently made to create a scientifically sound rubric of educational apps. So far, most studies found that so-called “educational apps” are often designed as to entertain children with sounds, images, and effects, with the result of distracting children rather than instructing them; moreover, these apps generally give too many choices to children, again resulting in distraction and lack of engagement. Importantly, even when effectively designed to engage, these apps lack true educational content.

Considering all the above findings, there is an urgent need for longitudinal studies focusing on the long-term effects of screen time and educational apps in very young children. This issue is indeed crucial not only to inform the caregivers’ rearing strategies, but also to orientate educational policies. We need to know better about this topic and we need to know it quickly, to catch up with the extremely fast pace of technological advancements.
